# Peri‐implantitis management in a patient with erosive oral lichen planus. A case report

**DOI:** 10.1002/ccr3.3617

**Published:** 2020-12-05

**Authors:** Rodrigo Martin‐Cabezas

**Affiliations:** ^1^ Swiss Dental Clinics Group Ardentis Clinique Dentaire Vevey Vevey Switzerland

**Keywords:** oral lichen planus, oral pathology, peri‐implant surgery, peri‐implantitis

## Abstract

Oral lichen planus did not seem to influence the peri‐implant healing. Oral lesions with malignant potential can mimic peri‐implantitis, and a biopsy should be performed in cases of progression or nonregression of the lesion after initial treatment.

## Graphical abstract

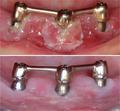

## 1 INTRODUCTION

The influence of oral lichen planus on peri‐implant treatment is unknown. This article reports the peri‐implant therapy in a patient affected by erosive oral lichen planus and peri‐implantitis. Success outcomes were obtained without recurrences after 1‐year follow‐up. Oral lichen planus did not seem to influence the peri‐implant treatment outcomes.

Peri‐implantitis is a pathological condition occurring in tissues around dental implants, characterized by inflammation in the peri‐implant connective tissue and progressive loss of supporting bone.^1^ This disease is characterized clinically by signs of inflammation, bleeding on probing and/or suppuration, increased probing depths, and/or recession of the mucosal margin in addition to radiographic bone loss compared with previous examinations.^2^ The prevalence of peri‐implantitis is 22% of subjects,^3^ and some risk factors have been related to peri‐implantitis development such as periodontitis (Odds ratio = 4.08)^4^ or poor plaque control (Odds ratio for plaque index ≥ 33% = 9.25).^5^ However, few studies analyzed the association between peri‐implantitis and oral lichen planus (OLP).^6^

Oral lichen planus is a mucocutaneous chronic immune‐mediated inflammatory disease affecting about 2% of the population. The most recognizable manifestation of OLP is the reticular variant that includes white lacey lines (Wickham striae) and hyperkeratotic papules and plaques. The malignant transformation of OLP lesions is continuously debated, the frequency ranges from 0% to 12.5%, and it is mostly reported in patients with atrophic or erosive OLP.^7^

Oral lichen planus is not considered an absolute contraindication for implant placement.^8^ In fact, the success rate does not differ from the general population and the manifestation of OLP is not influenced by implant placement.^6^ However, routine follow‐up examinations of peri‐implant tissues are specially recommended in patients with associated risk factors for malignancy such as OLP, because some oral lesions can mimic clinical appearance of peri‐implantitis.^1^, ^9^

Information about peri‐implantitis and OLP is scarce, and no reports have documented the peri‐implant treatment and healing in patients with OLP. Hence, the aim of this case report is to describe the peri‐implant therapy (nonsurgical and surgical) of a patient diagnosed with erosive OLP and peri‐implantitis, presenting gingival overgrowth and severe bone loss.

## 2 CLINICAL CASE

### 2.1 Case history/examination

**FIGURE 1:**
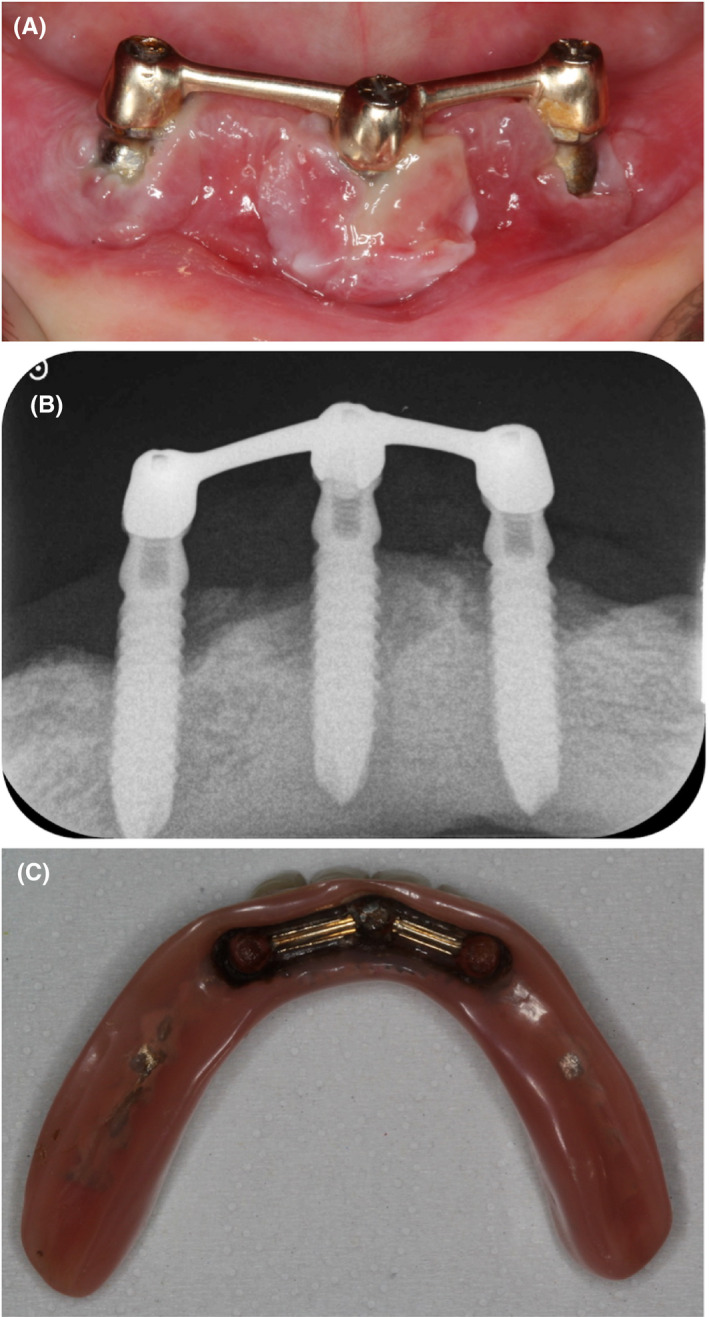
A) Clinical situation at baseline; B) X‐rays at baseline; C) Prosthesis

The patient was an 83‐year‐old Caucasian woman complaining about gum pain around dental implants. She had a history of chronic pulmonary embolia and takes 10 mg Xarelto (Rivaroxaban) per day. She was diagnosed with OLP more than 10 years ago but she had no specific treatment for this pathology. The intraoral examination showed a maxillary complete denture and a mandibular bar overdenture retained by three implants in position #43, #31, and #33 (Figure 1) placed 30 years ago. She reported attending recall visits with her general dentist, but she had never had a peri‐implant treatment. Both protheses were well adapted, and the patient was satisfied with them. Some weeks before the consultation, she had pain around the implants and the explantation of the three implants was proposed by her former dentist. The patient consulted for a second opinion as she wanted to keep her implants.

Oral lichen planus involved the retromolar and the buccal mucosa bilaterally, the lower lip, and the mucosa around all implants; indeed, gingival overgrowth and severe inflammation were present at the peri‐implant mucosa. Table 1 shows periodontal status at baseline with probing pocket depth (PPD) ranging from 5 to 11 mm and 100% of sites presenting bleeding on probing (BOP).

**TABLE 1:** Periodontal status at baseline

Lingual	BOP									
	PPD	8	7	9	11	11	13	9	6	6
**Implant**		**#43**			**#31**			**#33**		
Buccal	PPD	7	6	7	11	9	11	7	7	5
	BOP									

Abbreviations: BOP, Bleeding on probing; PPD, Probing pocket depth.

BOP positive sites are also in red color.

The radiographic examination showed circumferential peri‐implant bone loss around each implant. All implants were threaded with nontapered body. The bone loss around implants in position #43 and #33 was about one third of the implant length, while at the implant in position #31 the bone loss reached about 50% of the implant length (Figure 1).

### 2.2 Diagnosis

The presence of bleeding on probing, pocket depth ≥6 mm, and bone levels ≥3 mm apical of the most coronal portion of the intraosseous part of the implant, fulfilled the 2017 World Workshop on the Classification of Periodontal and Peri‐Implant Diseases and Conditions^2^ criteria for the diagnosis of peri‐implantitis for all the implants.

### 2.3 Treatment

#### 2.3.1 Initial peri‐implant therapy

The position and the prothesis design did not affect the access for oral hygiene so it was not modified. Oral hygiene instructions included the use of interdental brushes. Nonsurgical peri‐implant debridement was performed in one session under local anesthesia with ultrasonic devices adapted for implant surfaces and carbon curettes. 0.2% chlorhexidine mouthwash 3 times per day was prescribed for 7 days at the end of the initial phase.

#### 2.3.2 Peri‐implant re‐evaluation

**FIGURE 2:**
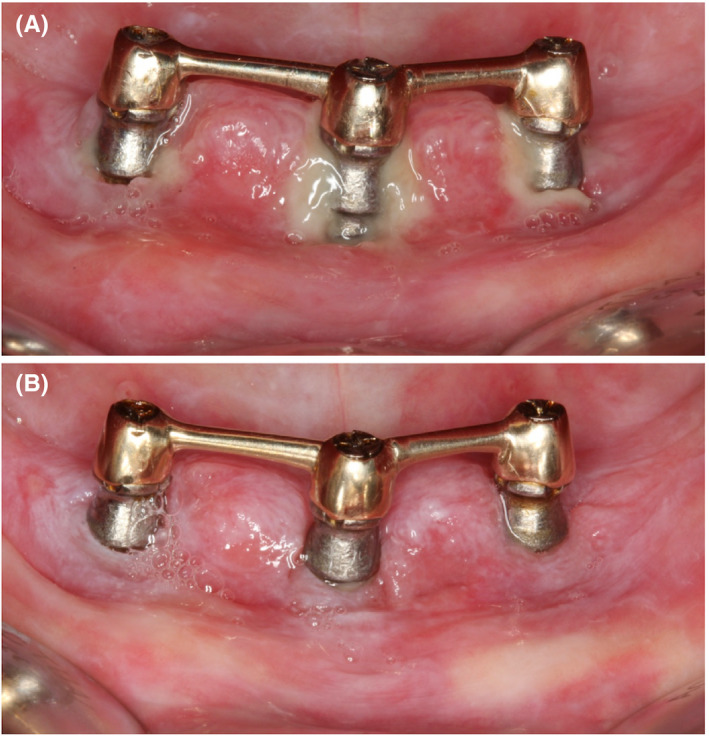
Follow‐up visits after nonsurgical debridement. A) Clinical view at 2 wk; B) Clinical view at 6 mo

Early clinical re‐evaluation was performed at 2 weeks to assess gingival healing. There was a regression of gingival overgrowth, but fibrin membrane was present at the gingival margin (Figure 2). Peri‐implant charting was reassessed at 3 months after nonsurgical therapy (Table 2), showing the presence of deep residual pockets. The surgical therapy was considered after confirming the safety of the procedure with her general practitioner, concerning the systemic medication.

**TABLE 2:** Periodontal status at 3‐month re‐evaluation after nonsurgical treatment

Lingual	BOP									
	PPD	8	6	9	11	11	11	7	7	7
**Implant**		**#43**			**#31**			**#33**		
Buccal	PPD	6	6	6	10	9	11	7	3	6
	BOP									

Abbreviations: BOP, Bleeding on probing; PPD, Probing pocket depth.

BOP positive sites are also in red color.

#### 2.3.3 Surgical peri‐implant therapy

Six months after initial therapy, resolution of gingival inflammation was achieved (Figure 2) and surgical treatment was performed to eliminate the remaining deep pockets and to repair the intrabony defects. As the suprastructure of the implants did not hinder access to perform the surgical treatment, it was not removed; moreover, it assured the stability of the prosthesis during the healing period.

**FIGURE 3:**
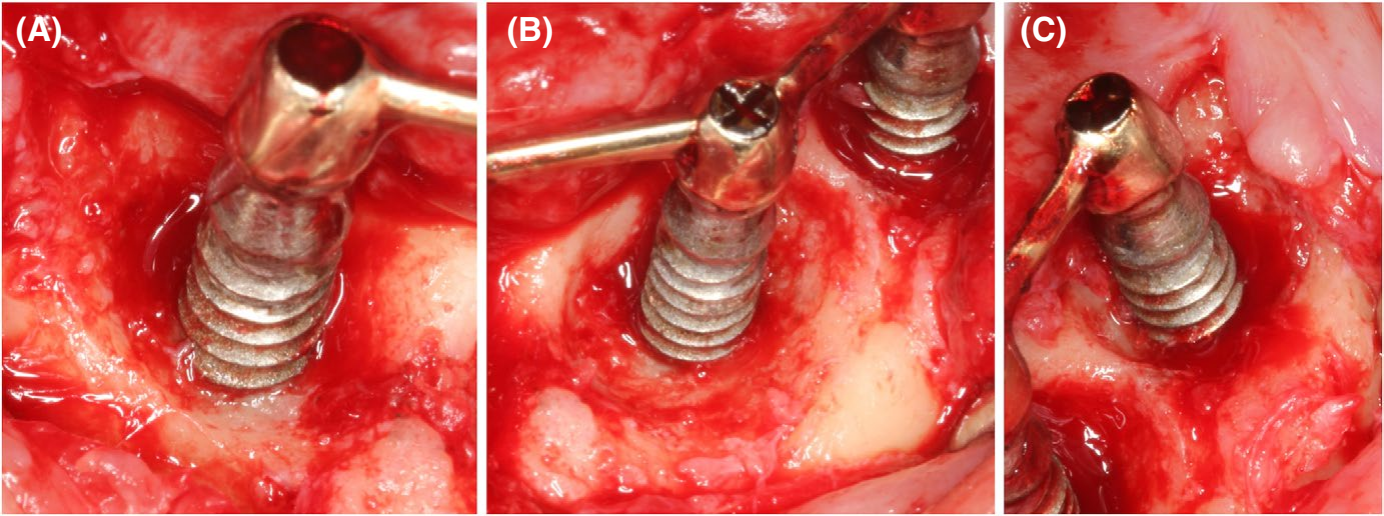
Surgical view of bone defects after granulation tissue debridement. A) Implant #43; B) Implant #31; C) Implant #33

After local anesthesia, crestal and para‐sulcular incisions were performed with a 15C blade extending distally from implants in position #33 and #43, and a mucoperiosteal flap was raised. After granulation tissue debridement, the bone loss was assessed. All implants had horizontal suprabony defects associated with large intrabony defects of about 2 mm depth. The intrabony component presented buccal dehiscence and circular bone resorption with maintenance of the lingual compacta for implants in position #43 (Class IIIb Grade M) and #31 (Class IIIb Grade A), while the intrabony defect around the implant in position #33 presented circular bone resorption with maintenance of the buccal and oral compacta (Class IIIc Grade M) (Figure 3).^10^

**FIGURE 4:**
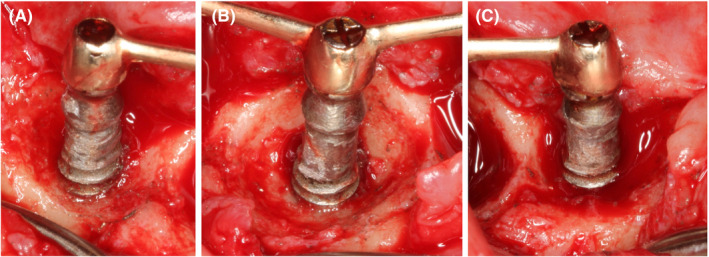
Surgical view after implantoplasty. A) Implant #43; B) Implant #31; C) Implant #33

**FIGURE 5:**
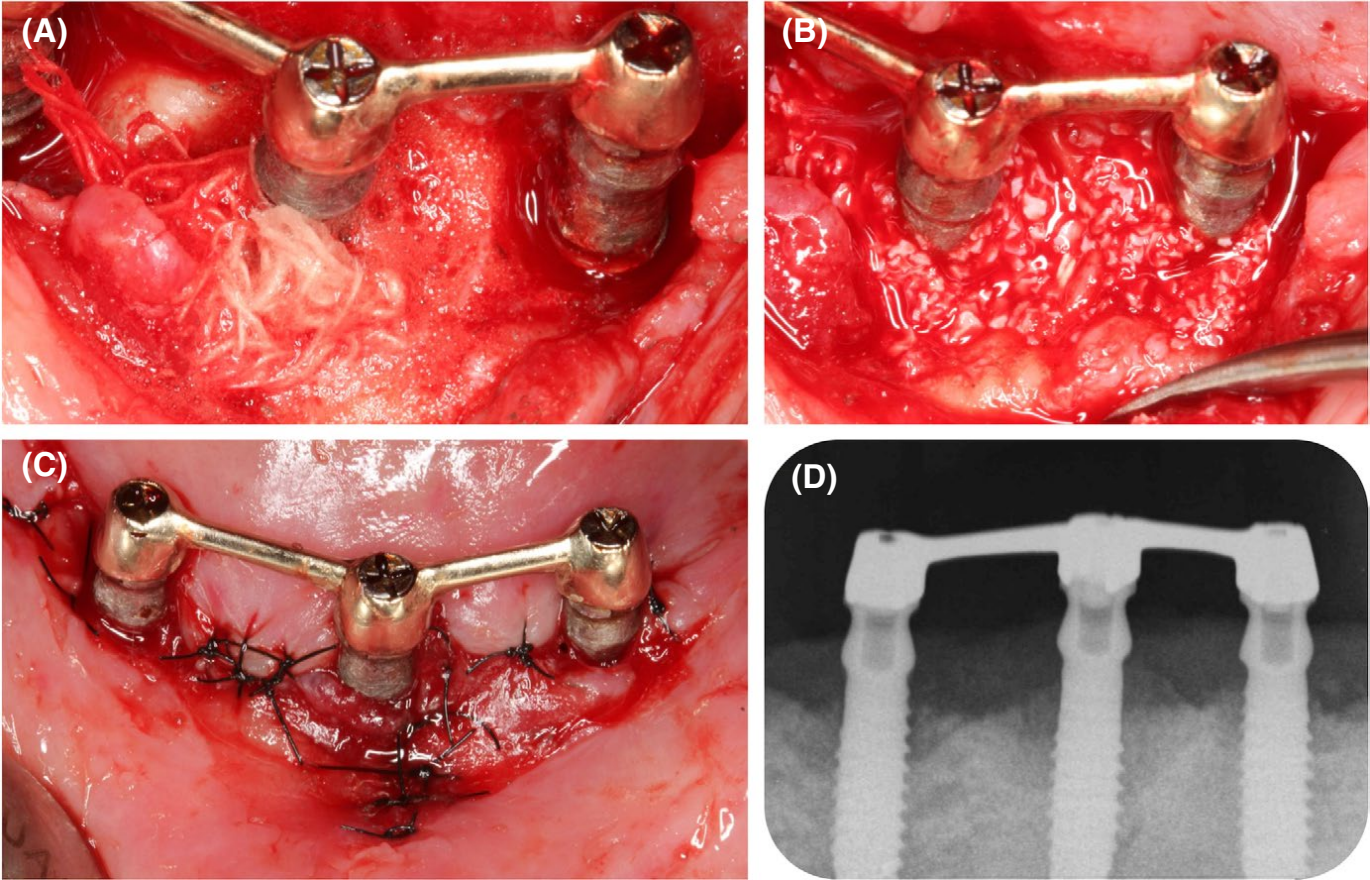
A) Implant surface decontamination with 3% H_2_O_2_; B) Bone substitute filling; C) Sutures; D) Postoperative x‐rays

Implantoplasty was performed with diamond burs to polish the implant threads of the suprabony part of the defects (Figure 4), and the implant surfaces were chemically decontaminated with 3% hydrogen peroxide (Figure 5A).^11^

In order to achieve repairing the intrabony part of the defect, a bone substitute (Bio‐Oss^®^ 0.25‐1 mm, Geistlich Pharma AG, Wolhusen, Switzerland) was placed without membrane.^12^ Interrupted nonresorbable sutures (5.0) were used to close the flap, and postsurgical radiograph was performed (Figure 5). Amoxicillin 1.5 g per day for 7 days and 0.2% chlorhexidine mouthwash 3 times per day for 14 days were prescribed.

### 2.4 Outcome and follow‐up

#### 2.4.1 Re‐evaluation after surgical treatment

**FIGURE 6:**
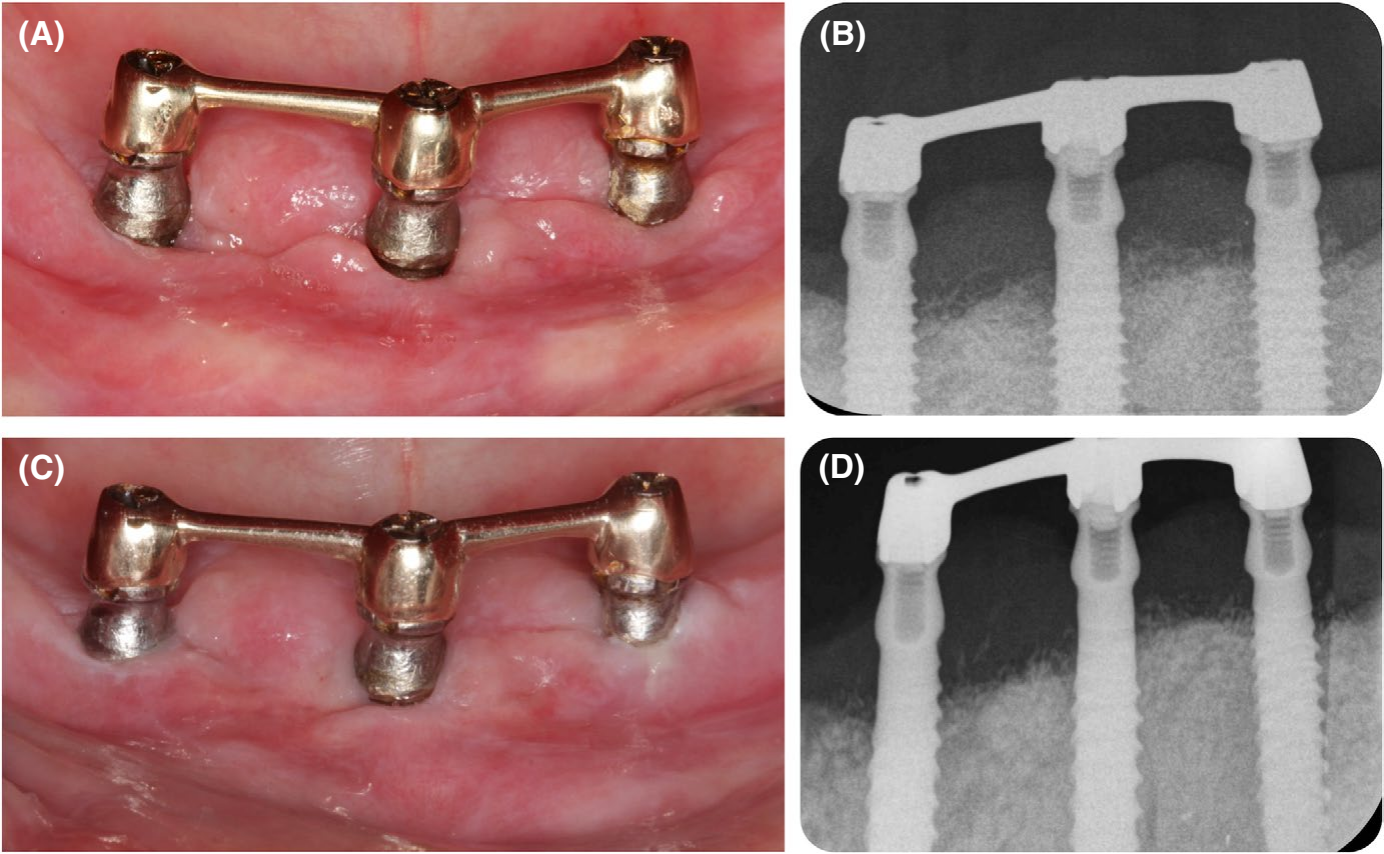
Follow‐up after surgical therapy. A) Clinical view at 1 mo; B) X‐rays at 1 mo; C) Clinical view at 6 mo; D) X‐rays at 6 mo

The patient was monitored every 3 months, and peri‐implant charting reassessed at 6‐month after peri‐implant surgery. Only moderate pockets remained (<5 mm), bleeding on probing decreased (Table 3) and radiographic examination revealed stability of bone levels with complete intrabony defects fill (Figure 6).

**TABLE 3:** Periodontal status at 6‐month re‐evaluation after surgical treatment.

Lingual	BOP									
	PPD	3	4	4	3	3	5	3	3	5
**Implant**		**#43**			**#31**			**#33**		
Buccal	PPD	3	4	4	4	4	4	4	4	5
	BOP									

Abbreviations: BOP, Bleeding on probing; PPD, Probing pocket depth.

BOP positive sites are also in red color.

#### 2.4.2 Maintenance phase

**FIGURE 7:**
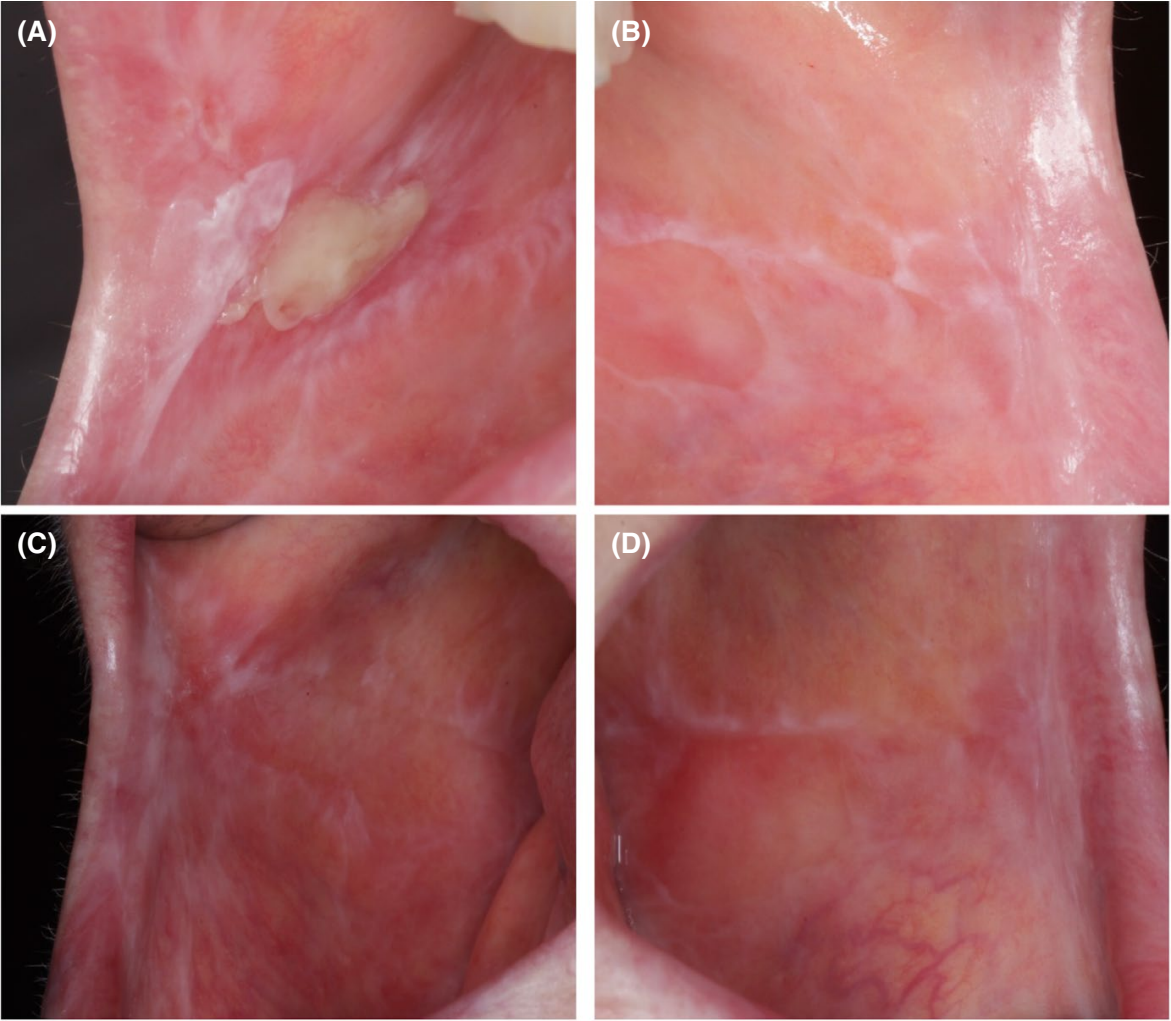
Cheek mucosa. A) Right side and B) left side at 6‐month re‐evaluation presenting ulcerations; C) Right side and D) left side at 1‐year re‐evaluation with complete resolution of the lesions

At 6 months, a maintenance protocol was established, including oral hygiene remotivation, supra‐mucosal biofilm removal, and debridement of residual pockets every 3 months.^13^ During the maintenance phase, some exacerbations of the OLP were present bilaterally at the cheek mucosa with ulcerations (Figure 7), which were treated with topical corticosteroid rinses.^14^ These exacerbations did not affect the peri‐implant conditions.

**FIGURE 8:**
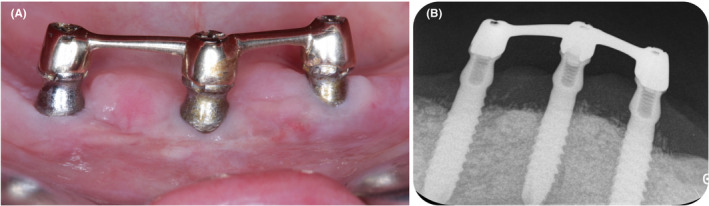
A) Clinical view; B) Radiographic examination 1 y after surgical treatment

One‐year after surgical peri‐implant therapy, clinical parameters (Table 4) and radiographic peri‐implant bone levels were reassessed (Figure 8). The clinical and radiographical outcomes confirmed a successful peri‐implant treatment with implant survival with no periodontal pockets ≥5 mm with concomitant bleeding on probing or suppuration or further bone loss.^12^

**TABLE 4:** Periodontal status at 1‐year re‐evaluation after surgical treatment.

Lingual	BOP									
	PPD	3	3	3	3	3	4	4	4	4
**Implant**		**#43**			**#31**			**#33**		
Buccal	PPD	3	3	3	3	3	3	4	3	4
	BOP									

Abbreviations: BOP, Bleeding on probing; PPD, Probing pocket depth.

BOP positive sites are also in red color.

## 3 DISCUSSION

This report shows the successful management of peri‐implantitis in a patient with erosive OLP and treated by peri‐implant nonsurgical and surgical therapy. Supportive therapy allowed results to be stable 1 year after surgical treatment.

It has been suggested that OLP‐affected mucosa may had an impaired capacity/ability to adhere to the implant surface, allowing the bacterial access/ingress to the peri‐implant surface which results in more peri‐implant infections.^15^, ^16^ However, in a prospective study, peri‐implantitis appeared in 10.7% of the implants and 27.7% of the patients with OLP without significant differences from the group of patients without OLP.^15^ Another study analyzing the peri‐implant conditions of patients with OLP, reported peri‐implantitis in 25% of implants, which did not differ significantly from the control group. The odds ratio (OR) for peri‐implantitis in the OLP group was 1.32 (0.81‐2.14, *P* = .257), concluding that OLP is not a risk factor for peri‐implantitis.^17^ A recent meta‐analysis^18^ found no differences when comparing patients with OLP and without OLP for the prevalence of or peri‐implant mucositis (19.6% vs. 22.7%, respectively) or peri‐implantitis (17.0% vs. 10.9%, respectively).

Moreover, dental implants do not influence OLP distribution.^19^ In this context, OLP is not considered a contraindication for implant placement, and survival and success rates of implants placed in OLP patients were 100% and 96.7%, respectively.^15^ However, there is a lack of information about the influence of OLP on the treatment and healing of peri‐implantitis. As biological plausibility, it should be considered that the presence of infection can act as an irritant, which triggers the immune reaction and results in worsening of oral mucosa lesions.^14^ Symptomatic erosive gingival lesions hinder adequate plaque control again exacerbating the immune response.^16^ In this context, the anti‐infective treatment of peri‐implantitis successfully resolves soft tissue inflammation,^2^ and reducing the chronic inflammation associated with both the peri‐implant and OLP lesions may effectively decrease the risk of malignant transformation.^7^

Regenerative procedures for peri‐implantitis, such as bone‐graft procedures with or without membranes, address the fill of the defect.^11^ Roos‐Jansåker et al,^20^ reported the clinical and radiographic outcomes of regenerative surgical treatment of peri‐implantitis comparing the use of bone substitute and membranes and the use of bone substitute alone. Both protocols resulted in an improvement and long‐term stability, without differences between both groups. The authors concluded that the use of a membrane did not add to the predictability or the bone fill. Similarly, a recent consensus report established that there is no evidence to support a specific material and no differences in the protocol of implant surface decontamination.^21^ The surgical treatment reported in this case combined the use of bone graft for the intrabony defect with implantoplasty of the exposed implant surface. The combination of implantoplasty and regenerative therapy has already been reported in advanced peri‐implantitis defects.^22^ The implantoplasty has been recommended in nonregenerative treatments allowing better clinical outcomes in terms of PPD and BOP reduction,^21^ and regeneration procedures have been recommended for circumferential and intrabony defects around implants.^23^

It is important to remember that some oral lesions that occur around implants, such as oral squamous cell carcinoma, metastases or giant cell, and pyogenic granuloma, may mimic peri‐implantitis.^1^ Differential diagnosis is determined clinically and a final diagnosis is only possible after histopathological analysis, especially in cases with precursor lesions of potential malignant transformation, such as erythroplakia, leukoplakia, proliferative verrucous leukoplakia, OLP, previous oral malignancy, extra‐oral malignancy, or in patients with risk habits as smoking (past or present) or alcohol abuse.^24^ The diagnosis of the OLP can be done clinically, and a biopsy can be used for confirmation of the diagnosis.^14^ In this context, a biopsy of the peri‐implant tissue is necessary in cases not responding to local treatment^9^, ^25^ or in cases that progress.^24^ As the patient had already been diagnosed with OLP ten years ago, a biopsy for confirmation of the diagnosis was not performed. Instead, the patient was monitored for regression of the lesion after effective therapy. After initial therapy, an early reassessment of peri‐implant health was performed showing a good response to initial treatment and no progression of the lesion. Otherwise, a biopsy and histopathological analysis should be done to reduce the risk of delayed diagnosis of malignant lesions.^24^

## 4 CONCLUSION

The OLP does not seem to influence the peri‐implant healing, even after peri‐implant regenerative surgery. Further studies including a larger number of implants and subjects are needed to confirm these results. Special attention should be paid to oral lesions with malignant potential as they can mimic the clinical appearance of peri‐implantitis.

## CONFLICT OF INTEREST

None declared.

## AUTHOR CONTRIBUTION

RMC: contributed to patient management, writing, and editing.

## ETHICAL APPROVAL

The article describes retrospectively a case report, and it does not contain any studies on human participants or animals. Therefore, no Ethics Committee was required.
